# Supplementary material for: From struggle to strength in African and Middle Eastern newcomers’ integration stories to Canada: A participatory health equity research study
Source: PLoS One. 2024 Apr 30;19(4):e0302591. doi: 10.1371/journal.pone.0302591 (PMC11060515; doi:10.1371/journal.pone.0302591)
Supplement: S1 Table — (PDF) [file pone.0302591.s001.pdf]

	Child Codes<br>Descriptive Theme	3 Representative<br>Excerpts	Description	All Extracted Excerpts<br>*(number of analysts out of 4 who coded for the quote)
Context	**Cold Winter Weather Conditions**<br>*Any comments about the effects of weather conditions, particularly during specific seasons like winter, including the impact on physical and mental well-being, daily routines and activities, and the overall environment.*	**Story 1:** "The cold of Canadian winter is something that no one really explains to you in ways that can prepare you. I remember leaving the plane and feeling like my skin was being ripped off"<br><br>**Story 10:** "The weather is what got under my skin due to adjusting from the warm climate to the cool and windy climate was really difficult for me. The new winter wardrobe is quite expensive as you need winter boots and a jacket which are very costly. Furthermore learning how to walk as the roads are very slippery took a lot of readjustment and learning how to do my daily activities (e.g the commute to and from school) despite the freezing conditions was not normal but now it is,<br><br>**Story 16:** No one prepares you for the kind of cold you experience here in Canada especially so when you're moving from a warm country....The depression that winter brings is one that has been spoken about quite a lot in the form of seasonal depression. I too, was a victim to this shortly after landing. I was particularly enraged by the 'fake sun' which is what I'd call it when the sun was out, shining despite it being -30 outside.	The cold winters make adapting to Canada more difficult and isolating for newcomers. Oftentimes it can take a toll on newcomers physically, emotionally and socially.<br><br>The winter seasons in Canada can feel very foreign to newcomers, especially those who come from more tropical weathers. With often gloomy weather, it can challenge one's mental health and make it more difficult to take care of ones self and settle in their new home. As the days are shorter and colder in the winter, it can often be more challenging for someone who isn't used to the weather conditions in Canada to get out of their home to excercise, get fresh air and meet new people. Therefore, achieving the basic needs and feeling like belonging during this time can seem more difficult for newcomers.	**Story 1 (4):** "I arrived to Canada … in the dead of winter. First of all, the cold of Canadian winter is something that no one really explains to you in ways that can prepare you. I remember leaving the plane and feeling like my skin was being ripped off"<br>**Story 2 (4):** "My biggest challenge while living in Canada is the cold, which is unavoidable and can really take a toll on one's mental health."<br>**Story 5 (4): "**My first winter was also a big adjustment, I was always warned that the winters in Canada were brutal and I definitely got to see and experience that."<br>**Story 6 (4):** "When I first arrived in Canada, I was immediately surprised by the winter! Coming from a hot and sunny country this was a big shock. Experiencing winter was and exiting and scary moment."<br>**Story 9 (3): "**Winter was also extremely cold over here which was shocking as well since I was used to living in temperatures around 20 °C all year long."<br>**Story 9 (1):** "My negative moments in Canada were adapting to the system, the responsibilities required for young students, the cold Canadian winter, and also the difficulty I faced when trying to make friends"<br>**Story 10 (4):** "My experience in Canada has been one of the best but the weather is what got under my skin due to adjusting from the warm climate to the cool and windy climate was really difficult for me. The new winter wardrobe is quite expensive as you need winter boots and a jacket which are very costly. Furthermore learning how to walk as the roads are very slippery took a lot of readjustment and learning how to do my daily activities (e.g the commute to and from school) despite the freezing conditions was not normal but now it is."<br>**Story 11 (4): "**The biggest challenge of all was adapting to the cold weather in Canada. Coming from a tropical climate, it took some time for me to get used to the cold winters and snowfall in Canada. It was also difficult for me to find appropriate winter clothing that would keep me warm during those cold months!"<br>**Story 13 (1):** "First, as every newcomer would say, the weather. When I look back probably the weather was not that bad because, crazy enough I got here in May, which means it was probably not cold at all for present me."<br>**Story 16 (3):** "No one prepares you for the kind of cold you experience here in Canada especially so when you're moving from a warm country like [country of origin in Africa]....The depression that winter brings is one that has been spoken about quite a lot in the form of seasonal depression. I too, was a victim to this shortly after landing. I was particularly enraged by the 'fake sun' which is what I'd call it when the sun was out, shining despite it being -30 outside."<br>**Story 16 (1)**: "My biggest challenge was moving and kind of having to adapt to a totally different environment- both in weather and in culture."
	**Unknown or Scary Migration Context:**<br>*There are lots of unknowns when moving to a new city as a newcomer, many of which can be scary. It requires courage to leave the comfort zone of familiarity.*	**Story 1**: "There is so much about Canada that no one never really warns you about. It's the unknown of it all that made my transition to Canada so daunting."<br><br>**Story 8:** "Before coming to Canada, I was scared about how my experience would turn out. Moving to a whole new country can be scary and you actually never know what to expect."<br><br>**Story 11:** "I had to leave behind my family, friends, and the life I had known for so long. "	Many newcomers discuss their transition to Canada as both thrilling and terrifying. Many find it scary not knowing what the future holds for them in the new country. Oftentimes, this is intensified when newcomers are seperated from their family and support systems back home; many describe not knowing where to obtain basic needs when first settling down. Beyond the fears, many also find the transition can be exciting when thinking about the new oportunities that may instill them in this new country. This instils a sense of hope for many newcomers and encourage them to be brave and step out of their comfort zone, exploring the unknowns of Canada.	**Story 1 (4):** "There is so much about Canada that no one never really warns you about. It's the unknown of it all that made my transition to Canada so daunting."<br>**Story 2 (4):** "Looking back, I can say my nervousness was less about leaving my home/safe place and more about the unknown that was awaiting me."<br>**Story 2 (1):** "Before coming to Canada, nervousness was the one feeling that keep persisting as I got ready to travel."<br>**Story 3 (3):** "This separation is a bit in conflict with our beliefs and traditions in my home country, where the youth might not become separated from families up until their marriage. ...The decision to immigrate to another country was an extremely tough one as I had been completely dependent on my parents mentally, emotionally, and financially. I was entirely hesitant about my future in the weeks prior to my flight, but my dreams told me I would find the way and become successful."<br>**Story 8 (4):** "Before coming to Canada, I was scared about how my experience would turn out. Moving to a whole new country can be scary and you actually never know what to expect."<br>**Story 8 (1):** "I didn't know what to buy and where to buy it from"<br>**Story 11 (2):** "I had to leave behind my family, friends, and the life I had known for so long. ";<br>**Story 13 (2):** "The best word to describe the first two years I spent in Canada would be <<blurry>>. I was definitely not prepared to what was waiting for me.";<br>**Story 15 (2):** "The transition from my homeland to Canada was the toughest process I could have gone through. Canada was a land of opportunities, but it was one that was completely foreign to us as we had no friends or family that could ease our integration."<br>**Story 16 (1):** I remember being back [home] a few days before I was set to travel and thinking about how life would look like, how cold it would be, would I look or seem all that different to people at school?"
	**School as key environment for adapation**<br>*Any comments regarding the atmosphere and surroundings in an educational setting, such as the environment in the classroom, school culture, relationships with classmates and teachers, and the overall experience of being a student.*	**Story 12:** What struck me on my first day of university was how little interest people had in talking and making new relations. Where I come from, university classes were very similar to high school classes...Classes here had more than 200 students in them, and so people just wanted to attend the class and leave. That definitely led to a sense of isolation at first, as I also had trouble knowing what to say and to whom. (Similar ideas in story 11)<br><br>**Story 13:** I had to start high school right away with people that didn't know anything about being new to a country because most of them were either first-generation Canadians or Canadians that immigrated a lot of years ago.<br><br>**Story 8:** However, the black community in my university was very welcomed and friendly. They showed me around campus and were there to answer any questions I had about the student experience in my university(how to get a bus pass, how to get a meal plan,etc).	Differences between education systems can make it challenging for newcomers to integrate into learning quickly; however, with resources and smaller communities (eg. international students associations) within schools can help improve newcomer integration experiences.<br><br>At first, integrating into a foreign school system can be very challenging for newcomers. Many newcomers discuss not knowing where they belong and feeling isolated with the large classroom sizes of the Canadian postsecondary system. However, with time and support from school resources, school is a great opportunity for newcomers to feel a sense of responsibility and foster their knowledge and skills towards persuing their ambitions. As well, school can often serve as a starting point for young newcomers to start establishing meaningful relationships with other young people in Canada. It is important to have resources available for newcomers within school systems to guide this transition, especially because some newcomers have discussed feeling challenged due to a lack of mental health support in the academic setting.	**Story 1 (1):** "other students are focused on their own lives"<br>**Story 2 (1):** "Switching from online to in person studying was a shock as well, I had to quickly develop social skills that have allowed me to build friendships with fellow students which I strongly believe is a pivotal part of one's academic journey.... I am well aware it [experiencing a positive integration process] is not always the case for the thousands of international students who struggle with mental health after moving here, reason why emphasis on providing a safe space for them is to be encouraged and promoted at all institutional levels, which ultimately benefits them academically."<br>**Story 4 (1):** "My education and previous work experience helped me land a good job. And I had a couple of friends here who supported me and gave me all the guidance I needed."<br>**Story 5 (4):** The one part I did not struggle with was getting used to college and my classes. I think it's because I was mentally prepared for what college life would be or look like and it was almost entirely right.<br>**Story 8 (2):** However, the black community in my university was very welcomed and friendly. They showed me around campus and were there to answer any questions I had about the student experience in my university(how to get a bus pass, how to get a meal plan,etc).<br>**Story 9 (1):** With the amount of students that need these [mental health] services, it was very difficult for me to obtain an emergency appointment with the therapists/psychologists at my university.<br>**Story 11 (3):** I also struggled with adjusting in campus, mode of schooling [in Africa] are quite different in some aspects which makes it seems difficult at first.<br>**Story 11 (2):** "Another positive moment was being able to pursue higher education opportunities in Canada that weren't available in Nigeria. With access to quality education, I was able to further my studies and gain valuable knowledge that has helped shape my career path today."<br>**Story 12 (4):** What struck me on my first day of university was how little interest people had in talking and making new relations. Where I come from, university classes were very similar to high school classes in that ... It was also generally a smaller group of students consisting of 30 students max. Classes here had more than 200 students in them, and so people just wanted to attend the class and leave. That definitely led to a sense of isolation at first, as I also had trouble knowing what to say and to whom.<br>**Story 13 (3):** "When I first got here as a refugee, I didn't have a house and had to go through the whole waiting process which means living in a shelter and on top of that I had to start high school right away with people that didn't know anything about being new to a country because most of them were either first-generation Canadians or Canadians that immigrated a lot of years ago. ... After that summer I started college and that's when I started feeling normal, I guess because I met people that were also new to the country and if there were not , at least college was new to all of us.<br>**Story 14 (2):** My parents wanted me to go to a French high school even though I've never studied in French. The first semester was very hard, I had to learn everything in french and also make friends at the new school.
	**Work**<br>*Any comments related to the atmosphere and conditions in the workplace, including aspects such as the culture, relationships with coworkers, etc.*	**Story 4:** "Even though I was born and raised in Lebanon, I was still categorized as a refugee with limited work opportunities." (In Canada) My education and previous work experience helped me land a good job."<br><br>**Story 11:** As an immigrant, it was hard for me to find employers who were willing to hire someone with no Canadian experience or references.<br><br>**Story 14:** I decided to help out and get a job but it was hard because I had no work experience in Canada.	Being a newcomer poses with unique circumstances that make it difficult to have the same work opportunities as those Canadian-born: limited work experiences and connections in Canada.<br><br>For many newcomers, it is important for them to find a stable source of income upon arrival to Canada in order to support themselves and/or their family. However, despite knowing English and having work exeperiences from back home, some newcomers experience difficulties with finding opportunities in Canada due to a lack of connections and people's prejudice. Oftentimes, work experiences from their home country does not translate the same to work experiences in Canada. Multiple newcomers discussed cashiering as their first source of income. Nevertheless, once newcomers find meaningful work experience in Canada, they often discuss the experience positively as it exposes them to the diversity of Canada and meet new people.	**Story 4 (2):** "Even though I was born and raised in Lebanon, [in Lebanon] I was still categorized as a refugee with limited work opportunities. I then appreciated that even though I come from a different background, Canada welcomed me and offered me new opportunities."<br>**Story 4 (1):** "My education and previous work experience helped me land a good job. ... COVID and working from home did not help ease my interactions with coworkers and potential friends."<br>**Story 6 (3):** Going to work made this a lot easier, as I met people from different backgrounds, cultures and religions. This has actually been amazing as I had never been exposed to much diversity.<br>**Story 11(3):** I planned on working [part] time so as to feed myself as I school but as an immigrant, it was hard for me to find employers who were willing to hire someone with no Canadian experience or references. After months of searching, I eventually found a job that allowed me to use my skills and experience from [Africa].<br>**Story 14 (4):** As I saw how my parents were struggling to get jobs and provide for us, I decided to help out and get a job but it was hard because I had no work experience in Canada but I kept applying and I got a job as a cashier. I would leave school and go straight to work. I didn't have any time for extracurricular activities or hanging around like other kids.
Integration Related Barriers	**Loneliness and Isolation**<br>*Comments regarding experiences of solitude and feelings of isolation (such as having limited social connections, spending a significant amount of time alone at home, etc.)*	**Story 1:** "The other thing that hit me so hard about this country is the loneliness that is so omnipresent. You can go a whole day without people ever acknowledging your existence."<br><br>**Story 3:** "Having no friends upon my arrival, my most pressing need at that early stage was to find someone that I could talk to."<br><br>**Story 6:** "One of the struggles I experienced as a newcomer was the constant feeling of feeling lonely."	Lack of family, friends, and community leads to loneliness and integration difficulties	**Story 1 (4):** "The other thing that hit me so hard about this country is the loneliness that is so omnipresent. You can go a whole day without people ever acknowledging your existence. ... I was used to a culture of my own people in which connecting with people, even strangers is so easy and comfortable"<br>**Story 3 (4):** "Having no friends upon my arrival, my most pressing need at that early stage was to find someone that I could talk to."<br>**Story 4 (1):** "I started comparing my current life in Canada to the one I had in Lebanon, wishing I would have my regular social interactions and comfort zone back."<br>**Story 6 (4):** "One of the struggles I experienced as a newcomer was the constant feeling of feeling lonely... Therefore, I didn't know anyone to talk to or ask for help and advice"<br>**Story 7 (2):** "I left my home country with two friends but we eventually parted ways... Coming from a country where it is very easy to get assistance from family and friends, it was hard being left alone to cater for all my needs. ... people were also busy with different responsibilities made it hard to meet up"<br>**Story 8 (2):** "Furthermore, when I came, I was scared about finding friends. Because I'm a timid person, I didn't know how to talk to people and had already in mind that I'll be by myself for a long time."<br>**Story 9 (2):** "I lived in... a small city [back in Africa] where everybody knows each other. After arriving in [Canada] to attend university, I was immediately shocked by the size of the city and also the diversity that exists in the community. It was difficult for me to make friends because the city was huge".<br>**Story 12 (4):** "Classes here had more than 200 students in them, and so people just wanted to attend the class and leave. That definitely led to a sense of isolation at first, as I also had trouble knowing what to say and to whom"<br>**Story 14 (3):** When I first arrived in Canada, it was very hard fitting in. Everybody knew each other and I felt like an outsider looking in.<br>**Story 15 (1):** "Canada was a land of opportunities, but it was one that was completely foreign to us as we had no friends or family that could ease our integration"
	**Language and Cultural Barriers**<br>*Any remarks or insights into the difficulties and obstacles faced when overcoming language barriers, such as the challenges of speaking, reading, writing, and comprehending. Any comments on the impacts of cultural differences in the process of transition and how they are affecting the individual.*	**Story 3:** "One of the first incidents I noticed was language disparities; it seemed that the way I learned English was not practical enough. Adversely, I discovered many of my classmates are bilingual. My mentality was moving on a downward slope, and I was fueled by the belief that my journey had failed."<br><br>**Story 4:** "I speak and understand English perfectly, but still I was not able to express and be myself around non-Arabic speaking individuals. My biggest challenge was trying to be myself and speak my "language" in English. I often found myself not participating in conversations because I was shy I would mispronounce a word."<br><br>**Story 11:** "When I first arrived in Canada, I felt overwhelmed by the language barrier. Although I had studied English in [Africa], it was still hard for me to understand the Canadian accent and slang. This made it difficult for me to communicate with people around me and make new friends."	Language and cultural differences creates a sense of disconnect	**Story 1 (1):** I was used to a culture of my own people in which connecting with people, even strangers is so easy and comfortable.<br>**Story 2 (3):** It was quite a challenge having to study in French , specifically the Canadian French which I was unable to grasp for a while but a year late I can gladly say it is not an issue anymore.<br>**Story 3 (4):** One of the first incidents I noticed was language disparities; it seemed that the way I learned English was not practical enough. Adversely, I discovered many of my classmates are bilingual. My mentality was moving on a downward slope, and I was fueled by the belief that my journey had failed.<br>**Story 4 (4):** "I speak and understand English perfectly, but still I was not able to express and be myself around non-Arabic speaking individuals. My biggest challenge was trying to be myself and speak my "language" in English. I often found myself not participating in conversations because I was shy I would mispronounce a word."<br>**Story 4 (3):** Coming from a completely different culture, it was very hard for me to know how to approach people appropriately, how to communicate in a casual tone, and when to add in some random jokes that will not sound "bad" or "awkward". I was also uncomfortable to join discussions because I was not well educated about the political system here, nor the economic situation, nor the basketball or hockey team players!<br>**Story 9 (2):** Everyone was also busy making connections and I was studying in an English-speaking university while back in [Africa], I studied in French under the Belgium education system.<br>**Story 9 (1):** "My positive moments were the cultural diversity I felt in Canada, work opportunities, and all the activities that I could do over here."<br>**Story 11 (4):** "When I first arrived in Canada, I felt overwhelmed by the language barrier. Although I had studied English in Nigeria, it was still hard for me to understand the Canadian accent and slang. This made it difficult for me to communicate with people around me and make new friends."<br>**Story 12 (1): "**Things improved as I began to understand the dynamics and interactions of students here. I had to adapt and change to how I thought I should be. I know realize that I should've been more myself, and that the right relationships would come by just being who I was, not who I thought I should be."<br>**Story 14 (2):** "My parents wanted me to go to a French high school even though I've never studied in French. The first semester was very hard, I had to learn everything in french and also make friends at the new school."<br>**Story 15 (4):** "Not only was the environment completely foreign in every aspect but we also had a significant language barrier to overcome. I was only comfortable in my native language, and I barely grasped the basic principles of French or English. Through several programs I was able to slowly bridge my language gap and start interacting with my peers."<br>**Story 16 (1):** "Moving and living in a different country for so long- 8 years for me now can prove to be hard to relate to those back home since you have missed so much since your departure. One then begins to question where you fit in. Some may consider you Canadian now because of your newfound openness or change in demeanour whilst others may consider you too African because of you lack of complete assimilation, down to your accent notwithstanding your culture and values."<br>**Story 16 (3):** "My biggest challenge was moving and kind of having to adapt to a totally different environment- both in weather and in culture."
	**Lack of Resources**<br>Any remarks or insights into the difficulties and obstacles faced when accessing resources before, during or after arrival to Canada and how it has impacted them	**Story 5:** "To apply for a VISA, I had to do in the country where I have citizenship. The application process was easy enough, but the wait to hear back from immigration was the longest pat. ... The experience would have been a lot better had immigration not taken such a long time to approve or deny my application because I would have been able to prepare for my trip more effectively."<br><br>**Story 9:** "With the amount of students that need these services, it was very difficult for me to obtain an emergency appointment with the therapists/psychologists at my university. This resulted in many depressed students and also students who committed suicide."	Community resources are needed to support the transition to Canada	**Story 3 (1):** I succeeded, and my new life started shaping, but I always have this question in my mind about how a person can address their mental health issues after immigration.<br>**Story 5 (2):** To apply for a VISA, I had to do in the country where I have citizenship. The application process was easy enough, but the wait to hear back from immigration was the longest pat. ... The experience would have been a lot better had immigration not taken such a long time to approve or deny my application because I would have been able to prepare for my trip more effectively.<br>**Story 9 (4):** "Another one of my challenges was accessibility to mental health services. With the amount of students that need these services, it was very difficult for me to obtain an emergency appointment with the therapists/psychologists at my university. This resulted in many depressed students and also students who committed suicide."<br>**Story 10 (2):** "The new winter wardrobe is quite expensive as you need winter boots and a jacket which are very costly."<br>**Story 11 (1):** "It was also difficult for me to find appropriate winter clothing that would keep me warm during those cold months!"<br>**Story 13 (2):** "When I first got here as a refugee, I didn't have a house and had to go through the whole waiting process which means living in a shelter."<br>**Story 15 (1):** "Through several programs I was able to slowly bridge my language gap and start interacting with my peers. ... The integration process would have been impossible if it wasn't for the access we had to community resources."<br>**Story 15 (1):** "Although the shelter offered some stability, it was a grim, crowded and highly restrictive place to call home."
	**Mental Health Issues**<br>*Comments regarding mental health and emotions experienced (such as low mood, depression, unhappiness, persistent worry, etc.)*	**Story 2:** "My biggest challenge while living in Canada is the cold, which is unavoidable and can really<br>take a toll on one's mental health."<br><br>**Story 3:** "My mentality was moving on a downward slope, and I was fueled by the belief that my journey had failed. After four months, stress, anxiety, and somehow depression overcame me. I went back to my home country. I lost considerable weight in just four months, and this made my parents feel nervous about my mentality."	Integration challenges can affect mental well-being<br>- Excerpts show that winter weather has a large impact	**Story 1 (1):** " There is this unspoken pressure of always seeming in control of our lives, that we must bear everything on our own."<br>**Story 2 (2):** My biggest challenge while living in Canada is the cold, which is unavoidable and can really take a toll on one's mental health.<br>**Story 3 (4):** My mentality was moving on a downward slope, and I was fueled by the belief that my journey had failed. After four months, stress, anxiety, and somehow depression overcame me. I went back to my home country. I lost considerable weight in just four months, and this made my parents feel nervous about my mentality.<br>**Story 9 (3):** Another one of my challenges was accessibility to mental health services. With the amount of students that need these services, it was very difficult for me to obtain an emergency appointment with the therapists/psychologists at my university. This resulted in many depressed students and also students who committed suicide.<br>**Story 7 (1):** "Luckily, being a believer really helped me overcome all struggles that may come with being alone, such as anxiety, depression, and bad moods."<br>**Story 8 (1):** "When I first arrived, moving into my new place was kind of stressful."<br>**Story 16 (1):** "The depression that winter brings is one that has been spoken about quite a lot in the form of seasonal depression. I too, was a victim to this shortly after landing".

Integration Related Facilitators	**Friendship**<br>*Any comments regarding relationships with friends, classmates, or coworkers and challenges or positive aspects of these interactions.*	**Story 13:** I made beautiful and wonderful friends who still play a big part in my life.<br><br>**Story 4:** I had a couple of friends here who supported me and gave me all the guidance I needed.<br><br>**Story 5:** It also helped that I already had friends from back home at my college which created a good work/life balance.	Friendships and connections help carry newcomers through challenges in new places.	**Story 1 (3):** It took me a very long time to have the courage to come out of my comfort zone and learn to connect with people here. I joined a boxing club and made many new friends there, friends from all over the world. I made friends from my job and my classes, and they helped me bear the stress of daily life.<br>**Story 2 (3):** I moved in with strangers who I now consider my family and who impacted my life in such a positive [way], providing a home abroad for me. Together with the fact that a couple of my closest friends from back home also came to live in Canada, I have not had to deal with a social lack that most international students go through<br>**Story 3 (1):** "Having no friends upon my arrival, my most pressing need at that early stage was to find someone that I could talk to. I was lost in the new lifestyle, and everything was like a shock to me."<br>**Story 4 (4):** I had a couple of friends here who supported me and gave me all the guidance I needed.<br>**Story 4 (1):** "Also, COVID and working from home did not help ease my interactions with coworkers and potential friends."<br>**Story 5 (3):** It also helped that I already had friends from back home at my college which created a good work/life balance.<br>**Story 7 (1):** " I had plans to meet my cousins on my second day here but wasn't able to call them to let them know where I'm staying and to figure out how we can meet. Hopefully, one of my roommates lent me her phone to call them. That same night, using my cousins' wifi while I was still at their place, I could text one of my friends my apartment address for her to come pick me up and help me with everything I had to do. She showed up the next day and helped me get a new phone number and food."<br>**Story 8 (2):** "However, the black community in my university was very welcomed and friendly. They showed me around campus and were there to answer any questions I had about the student experience in my university(how to get a bus pass, how to get a meal plan,etc)."<br>**Story 9 (2):** "It was difficult for me to make friends because the city was huge."<br>**Story 13 (3):** I made beautiful and wonderful friends who still play a big part in my life.
	**Family**<br>*Any comments on family ties, including the emotions and experiences associated with being apart from or missing family members.*	**Story 8:** "I'll always be thankful for having relatives in this country because they make my experience easier. When I have a problem or miss my family I can just call them and they'll always be there for me."<br><br>**Story 13:** "I had mixed feelings about the whole move but the fact that I was coming to a safer and stable country and was also about to get closer to a big part of my family (which included a nephew I hadn't met before) comforted me."<br><br>**Story 1:** "I learned that my family is always there for me and there is no shame in asking for help."	Strong familial support makes the transition to a new country more comfortable.	**Story 1 (4):** "I learned that my family is always there for me and there is no shame in asking for help."<br>**Story 2 (1):** " Everyday, family and friends came up to me with advice, warnings, life hacks and expectations for my "new adult life" journey that I was about to embark on especially since I was about to be the first one of my siblings to move away from home."<br>**Story 2 (3):** "It might come of as strange, but I barely felt homesick my entire time here and not because I did not miss my family/home but because the people in my life (both in my motherland & Canada) have provided immense love and support that carries me to this day."<br>**Story 3 (2):** "I lost considerable weight in just four months, and this made my parents feel nervous about my mentality. They talked to me a lot and convinced me that this was just the beginning. They told me that I am walking along a dark tunnel, and I should pass it to see the light outside."<br>**Story 5 (3):** "It was nice to find family here, it made the adjustment process a lot easier."<br>**Story 7 (1):** "A few days later, my uncle from [Canada] came to help me with the bank account and purchase some furniture I needed."<br>**Story 8 (1):** "I didn't know what to buy and where to buy it from but I'm lucky that I have relatives close to where I live who supported me throughout this period."<br>**Story 8 (4):** I'll always be thankful for having relatives in this country because they make my experience easier. When I have a problem or miss my family I can just call them and they'll always be there for me."<br>**Story 13 (3):** "I had mixed feelings about the whole move but the fact that I was coming to a safer and stable country and was also about to get closer to a big part of my family (which included a nephew I hadn't met before) comforted me."
	**Diversity**<br>*Any comments on the expression of their own or different cultures in Canada and their community.*	**Story 8:** However, I was also excited to discover a new culture and to become a little more independent.<br><br>**Story 16:** At the same time, moving here can be so exhilarating, exciting even because you get to start a fresh, get to experience new things, different people and cultures.<br><br>**Story 9:** My positive moments were the cultural diversity I felt in Canada, work opportunities, and all the activities that I could do over here.	Learning from new culture can help an individual grow, learn and gain a sense of identity	**Story 4 (1):** "I then appreciated that even though I come from a different background, Canada welcomed me and offered me new opportunities."<br>**Story 6 (4):** "Going to work made this a lot easier, as I met people from different backgrounds, cultures and religions. This has actually been amazing as I had never been exposed to much diversity. In addition, visiting parks, museums and different parts of the country allowed me to learn more about the Canadian culture and history."<br>**Story 8 (3):** "However, I was also excited to discover a new culture and to become a little more independent."<br>**Story 9 (4):** "After arriving in [Canadian city] to attend university, I was immediately shocked by the size of the city and also the diversity that exists in the community....My positive moments were the cultural diversity I felt in Canada, work opportunities, and all the activities that I could do over here."<br>**Story 10 (1):** "This country gave me a new perspective on how I see things as it was a change in environment."<br>**Story 11 (3):** "Despite all these challenges, there were also some positive moments when moving from [Africa] to Canada. One of them was being able to explore different cultures and meet people from all over the world. This gave me an opportunity to learn about different cultures and gain new perspectives on life."<br>**Story 12 (1):** "[A positive] being able to explore different cultures and meet people from all over the world. This gave me an opportunity to learn about different cultures and gain new perspectives on life."<br>**Story 16 (2):** "At the same time, moving here can be so exhilarating, exciting even because you get to start a fresh, get to experience new things, different people and cultures."
	**New Opportunities**<br>*Comments on different new experiences or opportunities they have had or gained in Canada*	**Story 11: "**Another positive moment was being able to pursue higher education opportunities in Canada that weren't available in Nigeria. With access to quality education, I was able to further my studies and gain valuable knowledge that has helped shape my career path today."<br><br>**Story 15:** "The programs offered by my community housing helped me to create social connections, opportunities for work, meaningful activities, and community participation."<br><br>**Story 1:** "I have recently learned to find ways to interact with people here. I joined a boxing club and made many new friends there, friends from all over the world."	The arrival to a new location brings new opportunities that can help facilitate the integration process.	**Story 1 (2):** "I joined a boxing club and made many new friends there, friends from all over the world."<br>**Story 4 (2):** I then appreciated that even though I come from a different background, Canada welcomed me and offered me new opportunities.<br>**Story 9 (2):** My positive moments were the cultural diversity I felt in Canada, work opportunities, and all the activities that I could do over here.<br>**Story 9 (1):** "Moreover, the services that the university offered for the transition between cultures and school systems really helped me a lot."<br>**Story 11 (3):** "Another positive moment was being able to pursue higher education opportunities in Canada that weren't available in [Africa]. With access to quality education, I was able to further my studies and gain valuable knowledge that has helped shape my career path today."<br>**Story 13 (1):** "After high school I got a summer job, which meant being somewhat financially stable, and that felt good because I couldn't depend on my parents completely as they were going through that change as well and it was sure tough on them even though they didn't dwell on it."<br>**Story 13 (2):** "After almost 6 years in Canada, I can say that I had a moment of confusion at the beginning but adapting was very easy and the opportunities I got and the people I met made it easier."<br>**Story 15 (3):** "Canada was a land of opportunities, but it was one that was completely foreign to us as we had no friends or family that could ease our integration. ... The programs offered by my community housing helped me to create social connections, opportunities for work, meaningful activities, and community participation."<br>**Story 16 (1):** "At the same time, moving here can be so exhilarating, exciting even because you get to start a fresh, get to experience new things, different people and cultures."
Resolutions and Learning	**Needing Time to Adapt**<br>*Any comments on the process of coming to Canada and how their experience has changed or not throughout time.*	**Story 1:** It took me a very long time to have the courage to come out of my comfort zone and learn to connect with people here. I have recently learned to find ways to interact with people here.<br><br>**Story 12:** "Things improved as I began to understand the dynamics and interactions of students here. I had to adapt and change to how I thought I should be."<br><br>**Story 13:** "After almost 6 years in Canada, I can say that I had a moment of confusion at the beginning but adapting was very easy and the opportunities I got and the people I met made it easier."	Newcomers express the willingness and ability to adapt to new lifestyles.<br><br>Many newcomers face challenges as they navigate a new culture, language, social norms, lifestyle, and environment. Time, patience, and the willingness to accept change allowed many newcomers to embrace the new environment. In several stories, participants highlighted that their initial experiences in Canada were not always as they had hoped and that it typically required a period of adjustment. Many expressed the need to adapt and change their perspectives to improve their well-being and feel more comfortable in their new environment. (Learning, Coping, Core-self)	**Story 1 (4):** It took me a very long time to have the courage to come out of my comfort zone and learn to connect with people here. I have recently learned to find ways to interact with people here.<br>**Story 2 (1):** " It was quite a challenge having to study in French, specifically the Canadian French which I was unable to grasp for a while, but a year later I can gladly say it is not an issue anymore."<br>**Story 4 (2):** "In one year, Canada gave me a sense of belonging that I did not ever feel during my 28 years living in Lebanon. Adapting to the language, culture, and people is still something I work on every day. It is part of me growing as a person and opening up to this new side of the world. It is still not an easy process."<br>**Story 6 (3):** "Another challenge I faced was coming to the realization that change is not an easy process. Change is a journey with different paths."<br>**Story 7 (1):** "Coming from a country where it is very easy to get assistance from family and friends, it was hard being left alone to cater for all my needs, including groceries, cooking, cleaning, and many more, but I eventually managed to adapt to a new lifestyle real quick."<br>**Story 10 (1):** "Furthermore learning how to walk as the roads are very slippery took a lot of readjustment and learning. learning how to do my daily activities (e.g the commute to and from school) despite the freezing conditions was not normal but now it is"<br>**Story 11 (3):** "Coming from a tropical climate, it took some time for me to get used to the cold winters and snowfall in Canada. ... I also struggled with adjusting in campus, mode of schooling in [Africa] are quite different in some aspects which makes it seems difficult at first."<br>**Story 12 (2):** "Not only did I have to adjust to a new academic system, I also had to adapt to a new country and culture, all the while living on my own for the first time. ...Things improved as I began to understand the dynamics and interactions of students here. I had to adapt and change to how I thought I should be."<br>**Story 13 (3):** "After almost 6 years in Canada, I can say that I had a moment of confusion at the beginning but adapting was very easy and the opportunities I got and the people I met made it easier."
	**Developing New Strategies/Habits**<br>*Comments regarding perseverance and learning or practicing strategies to overcome obstacles.*	**Story 1:** "I have recently learned to find ways to interact with people here. ... I learned that my family is always there for me and there is no shame in asking for help."<br><br>**Story 6:** "However, with time I decided to engage and be more active in my new reality. This was achieved by making the effort of going to community centers that help newcomers. The community centers were helpful as they provided many helpful resources that helped me till this day. I also had to come out of my comfort zone and engage with my new community."<br><br>**Story 9:** "Regarding the strategies that I used in order to integrate in this country and new city, I tried to ask questions to people who has lived here for several years so that I could discover new activities to do, get out of my comfort zone even if the activity does not interest me at first, and also to visit other cities in Canada."<br><br>**Story 15:** "Through several programs I was able to slowly bridge my language gap and start interacting with my peers."... "The integration process would have been impossible if it wasn't for the access we had to community resources."	Community involvement, whether through hobbies or programs offered on campus, helps newcomers connect with others and feel more at home.<br><br>Often, individuals immigrating to Canada arrive without companionship or the support of an extensive social network. As a result, they are forced to learn and develop new skills and habits. One of the biggest challenges highlighted in the stories was connecting with others and feeling a sense of belonging. As a way to adapt, one newcomer mentioned that joining a boxing club helped them make more friends. In contrast, others found that involvement at community centers helped them access resources and engage further with others. Essentially, many newcomers emphasized that they had to proactively develop strategies and habits that would enable them to integrate smoothly with their community and social circle. (Coping, Belonging, Core-Self)	**Story 1 (1):** "I have recently learned to find ways to interact with people here. I joined a boxing club and made many new friends there, friends from all over the world. I made friends from my job and my classes, and they have helped me bear the stress of daily life. I learned that my family is always there for me and there is no shame in asking for help."<br>**Story 1 (3):** "To be happy one must be able to learn to seek our communities in happiness and in sadness."<br>**Story 2 (4):** "Taking vitamins, prioritizing a healthy sleep schedule, avoiding 8am classes are all strategies I use till this day which have personally helped me both mentally and academically."<br>**Story 4 (2):** "Every day I feel vulnerable when I put myself out there and when I force myself to engage with new people, make new friends, and learn about the different cultures. This is how I'm growing out of my comfort zone and appreciating myself more."<br>**Story 5 (1):** "Plus, I tried my best to stay on top of my school work to keep from getting too overwhelmed. It also helped that I already had friends from back home at my college which created a good work/life balance."<br>**Story 6 (3):** "However, with time I decided to engage and be more active in my new reality. This was achieved by making the effort of going to community centers that help newcomers. The community centers were helpful as they provided many helpful resources that helped me till this day. I also had to come out of my comfort zone and engage with my new community."<br>**Story 9 (3):** Regarding the strategies that I used in order to integrate in this country and new city, I tried to ask questions to people who has lived here for several years so that I could discover new activities to do, get out of my comfort zone even if the activity does not interest me at first, and also to visit other cities in Canada.<br>**Story 15 (2):** "Through several programs I was able to slowly bridge my language gap and start interacting with my peers. ... The integration process would have been impossible if it wasn't for the access we had to community resources."
	**Gained Resilience**<br>*Comments on overcoming challenges and growth or lessons they have gained*	**Story 3:** "They talked to me a lot and convinced me that this was just the beginning. They told me that I am walking along a dark tunnel, and I should pass it to see the light outside. I started again, and I came back to Canada once more. My ambition and my goals energized me to be hopeful this time."<br><br>**Story 12:** "I do believe that all those experiences, for better or worse, shaped me into the person I am today. I learned a lot about myself over the last few years, and so despite the challenges and the difficulties, I wouldn't change much. It taught me resilience and endurance, and that it was fine to be on your own for a while. It also taught me that I am much stronger than I believed I was, and those lessons I will carry with me wherever I go."<br><br>**Story 14:** "Overall I'm very grateful for all the struggle I went through cause it taught me hard work and not depending on others."	Perseverance and Resilience: Transforming hardship into opportunity.<br><br>Support from family, the resolution to persevere, and the ability to learn from adversity enabled many newcomers to cultivate diligence and independence, transforming them into a better version of themselves. (Learning, Core-Self)	**Story 3 (4):** "I started again, and I came back to Canada once more. My ambition and my goals energized me to be hopeful this time."<br>**Story 4 (2):** "Adapting to the language, culture, and people is still something I work on every day. It is part of me growing as a person and opening up to this new side of the world. It is still not an easy process. Every day I feel vulnerable when I put myself out there and when I force myself to engage with new people, make new friends, and learn about the different cultures. This is how I'm growing out of my comfort zone and appreciating myself more."<br>**Story 6 (1):** "I also had to come out of my comfort zone and engage with my new community. Going to work made this a lot easier, as I met people from different backgrounds, cultures and religions."<br>**Story 7 (1):** "Luckily, being a believer really helped me overcome all struggles that may come with being alone, such as anxiety, depression, and bad moods."<br>**Story 10 (1):** "This country gave me a new perspective on how I see things as it was a change in environment."<br>**Story 11 (2):** "Overall, moving from [Africa] to Canada has been both challenging and rewarding at the same time. Despite all the struggles I faced along the way, I'm grateful for all the experiences that have shaped who I am today!"<br>**Story 12 (4):** I do believe that all those experiences, for better or worse, shaped me into the person I am today. I learned a lot about myself over the last few years, and so despite the challenges and the difficulties, I wouldn't change much. It taught me resilience and endurance, and that it was fine to be on your own for a while. It also taught me that I am much stronger than I believed I was, and those lessons I will carry with me wherever I go.<br>**Story 13 (1):** "After almost 6 years in Canada, I can say that I had a moment of confusion at the beginning but adapting was very easy and the opportunities I got and the people I met made it easier. I am planning on starting my bachelor's degree in September after taking a 2-year break after my college graduation, I am very much used to the temperatures and enjoy May's weather."<br>**Story 14 (3):** Overall I'm very grateful for all the struggle I went through cause it taught me hard work and not depending on others.<br>**Story 16 (1):** "Some may consider you Canadian now because of your newfound openness or change in demeanour whilst others may consider you too African because of you lack of complete assimilation, down to your accent notwithstanding your culture and values."
	**Belief in a better future (hope)**<br>*Messages of hope and positivity after overcoming their fear or towards the future*	**Story 1: "**To be happy one must be able to learn to seek our communities in happiness and in sadness. One should not bear the world alone, others are always there for support, one must simplremember to call for them."<br><br>**Story 4:** "In one year, Canada gave me a sense of belonging that I did not ever feel during my [many] years living in Lebanon. Adapting to the language, culture, and people is still something I work on every day. It is part of me growing as a person and opening up to this new side of the world."... "I hope that in a few years, I will be able to confidently say "Canada is home".<br><br>**Story 16:** With the advent of summer, I noticed such a change in aura and number of people outside. People were happy. This was the canada I had been looking forward to coming to back when I was in high school. So much to do, so much to see and experience, all with the same weather that I was used to.	Opportunities, support, and belonging bring hope and happiness to Newcomers in Canada.<br><br>The accessibility and ability to ask for support, a sense of belonging, and the endless opportunities that once seemed impossible to reach, bring happiness and hope to many Newcomers. (Core-Self)	**Story 1 (3):** "To be happy one must be able to learn to seek our communities in happiness and in sadness. One should not bear the world alone, others are always there for support, one must simplremember to call for them."<br>**Story 3 (3):** I started again, and I came back to Canada once more. My ambition and my goals energized me to be hopeful this time. I succeeded, and my new life started shaping.<br>**Story 4 (1):** "In one year, Canada gave me a sense of belonging that I did not ever feel during my [many] years living in [the Middle East]. Adapting to the language, culture, and people is still something I work on every day. It is part of me growing as a person and opening up to this new side of the world."<br>**Story 4 (4):** "I hope that in a few years, I will be able to confidently say "Canada is home"."<br>**Story 7 (3):** "So far, I got used to this new lifestyle and I am willing to help newcomers because I was once one and know how it can get hard to adapt to a new environment."<br>**Story 11 (2):** Overall, moving from [Africa] to Canada has been both challenging and rewarding at the same time. Despite all the struggles I faced along the way, I'm grateful for all the experiences that have shaped who I am today!<br>**Story 13 (1):** After almost 6 years in Canada, I can say that I had a moment of confusion at the beginning but adapting was very easy and the opportunities I got and the people I met made it easier.<br>**Story 16 (1):** With the advent of summer, I noticed such a change in aura and number of people outside. People were happy. This was the canada I had been looking forward to coming to back when I was in high school. So much to do, so much to see and experience, all with the same weather that I was used to.
