# Supplementary material for: From struggle to strength in African and Middle Eastern newcomers’ integration stories to Canada: A participatory health equity research study
Source: PLoS One. 2024 Apr 30;19(4):e0302591. doi: 10.1371/journal.pone.0302591 (PMC11060515; doi:10.1371/journal.pone.0302591)
Supplement: Attachment — Submitted filename: Response_Letter.pdf [file pone.0302591.s003.pdf]

Dear reviewers,

Many thanks for the constructive feedback you provided. We integrated the comments in the new submitted manuscript.

Thanks for your kind consideration,
Corresponding author,
Dr. Kevin Pottie

1. **It is with interest that I have read this manuscript in its revised version. This research adds important understanding to the challenges that face newcomers from Africa and the Middle East and points out opportunities to facilitate their integration and support building their resilience. The results set a foundation for future research and initiatives to improve health equity and social services tailored to this marginalized population.**

   The authors utilized appropriate relevant literature to support their approach, including those that justify their predetermined age range of recruits between 18-30. Moreover, the need to run such research and its implication for improving the welfare of such communities was properly described, which made the objective clear. The selected type of research was also properly justified based on previous literature.

   The authors clearly described their methodology as well as their strategic involvement of newcomer researchers to help build the "trust" needed for the input of the participants. However, in response to the first reviewer inquiry regarding any prompts or instructions given to the participants, the authors explained that "Participants were provided with an e-learning module on storytelling, which outlined prompts and story examples to guide participants. We have included this e-learning module in our supporting information" I would suggest mentioning this in the methodology as it gives valuable information on how the stories were collected from the participants, which would guide future research.

   Response: Thank you for your helpful review. We have incorporated your suggestion in the methodology of our revised manuscript.

2. **In line [115], the statement is an exact repetition of the opening statement in line [86]. If the authors find the need to mention the approach again in that section, I suggest rephrasing it to avoid redundance.**

Response: Integrated in the manuscript.

3. **In the Results section, the added table was useful. I would suggest replacing “Count” by “Frequency (n=18)” and replace “Frequency” with “Percentage”. Also, in the table section (Highest Level of Education Completed), the total is 19 which suggests an error to be corrected.**

   Response: Thank you for your recommendation and for bringing this error to our attention. Revisions have been made to the manuscript.

4. **In lines [342-344] of the Discussion section, I found the statement a bit unclear. I had to read it several times to understand that the authors found that the amount of literacy, trust, resilience, and self-confidence were attributes that could enable emerging leaders. I suggest a simple rephrasing to avoid the unclarity of an important statement.**

   Response: Integrated in the manuscript.

5. **In lines [426-435], the authors discuss the limitations of their research, including the recruitment process. It might be useful if they can suggest recommendations to overcome this limitation in future research based on their experience, which would be beneficial for future researchers building on their methodology.**

   Response: Thank you for your recommendation. We have integrated this point in the manuscript.
